# *Plasmodium falciparum* leucine-rich repeat 5 disruption alters the transcription progression during asexual and sexual stage development

**DOI:** 10.1128/msphere.00186-26

**Published:** 2026-06-15

**Authors:** Camilla V. Pires, Chengqi C. Q. Wang, Min Zhang, Chiara Micchelli, Prem Prakash, Jenna Oberstabler, Julian C. Rayner, Thomas D. Otto, John H. Adams

**Affiliations:** 1 Center for Global Health and Interdisciplinary Research, Department of Global, Environmental, and Genomic Health Sciences, College of Public Health, University of South Florida 7831, Tampa, Florida, USA; 2 Department of Molecular Medicine, Morsani College of Medicine, University of South Florida 7831, Tampa, Florida, USA; 3 Cambridge Institute for Medical Research, University of Cambridge 2152, Cambridge, United Kingdom; 4 Institute of Infection, Immunity, and Inflammation, College of Medical, Veterinary and Life Sciences, University of Glasgow 3526, Glasgow, United Kingdom; 5 Bernhard Nocht Institute for Tropical Medicine 14888, Hamburg, Germany; 6 University of Hamburg 14915, Hamburg, Germany; University at Buffalo-Downtown Campus, Buffalo, New York, USA

**Keywords:** *Plasmodium falciparum*, single-cell RNAseq, leucine-rich repeat 5

## Abstract

How *Plasmodium falciparum* responds to stresses such as antimalarial drug pressure and uses them to trigger developmental decisions such as the production of sexual gametocytes remains poorly understood. Here, we investigated the function of the leucine-rich repeat protein LRR5 (PF3D7_1432400). Previous genome-wide *piggyBac* mutagenesis established that disruptions of LRR5 increase sensitivity to artemisinin and heat-shock and enhance gametocyte production. Using an optimized single-cell RNA sequencing approach applied to unsynchronized parasite cultures, we profiled transcriptional heterogeneity across the intraerythrocytic developmental cycle in wild-type NF54 parasites and an isogenic LRR5 *piggyBac* mutant (LRR5pB). Disruption of LRR5 reshaped the parasite transcriptome across multiple stages, with marked downregulation of pathways involved in protein folding, protein export, and host-cell remodeling, including Maurer’s cleft components, the PTEX export machinery, and the exported chaperone HSP70-x. In contrast, cytoplasmic heat-shock proteins and translation-associated genes were upregulated, suggesting a compensatory stress response. Notably, LRR5pB parasites displayed premature activation of gametocyte-associated transcriptional programs, including elevated expression of the early gametocyte marker Pfs16, which was confirmed at the protein level. Pseudotime analysis further revealed accelerated transcriptional progression with mistimed expression of trophozoite-, merozoite-, and gametocyte-associated gene clusters. Together, these findings suggest LRR5 as a link between proteostasis, host-cell remodeling, and developmental timing in *P. falciparum*.

**IMPORTANCE:**

Malaria parasites must survive environmental stress while deciding whether to continue asexual replication in the blood stream or instead commit to sexual gametocytes for transmission. Understanding how these processes are coordinated is critical for developing new strategies to block both disease and transmission. In this study, we used single-cell transcriptomics to examine how disruption of the *P. falciparum* protein LRR5 alters parasite development. By analyzing individual parasites from unsynchronized cultures, we uncovered hidden changes in stress-response pathways, host-cell remodeling, and early activation of genes linked to sexual development. Our findings show that loss of LRR5 weakens the parasite’s ability to manage stress while prematurely priming parasites for gametocyte formation. These results suggest LRR5 as a link to stress adaptation to transmission potential.

## INTRODUCTION

Malaria is a deadly mosquito-transmitted infectious disease caused by *Plasmodium* parasites. Malaria mortality and morbidity rates have decreased globally over the past two decades, but since the COVID-19 pandemic, this trend has reversed, and in 2020, there were approximately 627,000 deaths and 241 million infections attributed to malaria (1). The previous decline in cases was driven, in part, by effective treatments, mainly involving artemisinin and its derivatives combined with a partner drug, an approach known as artemisinin combination therapy (1, 2). Reduced efficacy of artemisinin, linked to decreased susceptibility of ring-stage parasites (3, 4) and recrudescence and treatment failure in patients (5–9), is now widespread globally, and alternative therapeutics and a deeper understanding of artemisinin responses in the parasite are both urgently needed.

Progress in deciphering *Plasmodium* gene function has historically been slow, as traditional gene-by-gene approaches are labor-intensive and complicated by the parasite’s highly AT-rich genome (~82%), which limits the efficiency of targeted editing tools such as CRISPR. Although individual studies have provided valuable insights—particularly since the completion of the *P. falciparum* genome two decades ago—the limited throughput of targeted methods, combined with the parasite’s evolutionary distance from model eukaryotes, has restricted systematic functional exploration. Consequently, more than 90% of *P. falciparum* genes remain untested by targeted gene studies, and until recently approximately 35% of its ~5,500 genes still lacked experimental functional annotation (10, 11). Developing high-throughput functional genomic approaches is, therefore, critical for accelerating the discovery of essential parasite pathways and novel therapeutic targets against this adaptable pathogen.

We previously applied random *piggyBac* transposon insertional mutagenesis to identify genes essential for *P. falciparum* blood-stage survival, generating a near-saturation mutant library containing approximately 38,000 single-insertion mutants (12). This large-scale effort defined 2,680 genes as essential for asexual blood-stage growth, including nearly 1,000 *Plasmodium*-conserved genes of unknown function. Building on this resource, the *piggyBac* mutant (*pB*-mutant) library has proven to be a powerful platform for the systematic functional annotation of the *P. falciparum* genome through genome-wide phenotypic screens. Using forward-genetic approaches based on random *piggyBac* mutagenesis (13–19), we have successfully identified genes essential or dispensable for asexual (12) and gametocyte development (16). These studies revealed that *P. falciparum* parasites exploit innate febrile-response mechanisms to modulate artemisinin sensitivity (17). Among the genes identified, PF3D7_1432400, encoding a leucine-rich repeat protein (LRR5), stood out as disruption led to increased sensitivity to heat shock and artemisinin derivatives (dihydroartemisinin [DHA] and artesunate) as well as increased gametocyte conversion rates, referred to as a hyper-producer phenotype. In *Plasmodium* spp., LRR-containing proteins facilitate protein–protein interactions and regulate the cell cycle through modulation of phosphatase activity (20, 21). For example, in *P. berghei*, the LRR protein PbLRR1 downregulates protein phosphatase 1 (PP1) activity and is required for efficient oocyst development (22), whereas in *P. falciparum*, LRR1 binds to serine/threonine phosphatase type 1 and inhibits parasite growth *in vitro* (23). Although *P. falciparum* encodes 14 LRR-containing proteins (24), the specific role of LRR5 in the asexual blood-stage cycle and gametocyte development remains unknown.

Using unsynchronized *P. falciparum* cultures single-cell RNA sequencing (scRNA-seq), we profiled individual parasites from the isogenic wild-type NF54 line and the LRR5pB mutant to investigate how LRR5 disruption affects the parasite’s response to artemisinin, stress, and developmental progression. The scRNA-seq data revealed altered expression of heat-shock protein genes, as well as genes involved in protein export and host-cell remodeling, together with the premature activation of early gametocyte markers, including the parasitophorous vacuole membrane protein Pfs16. These transcriptional signatures align with the phenotypic characteristics of the mutant—heightened sensitivity to febrile and drug stress, reduced growth rate, and increased gametocyte production—and are further supported by protein-level validation of Pfs16 upregulation. Collectively, these findings reveal associations between LRR5 disruption and altered proteostasis, host-cell remodeling, and sexual differentiation in *P. falciparum*.

## RESULTS

### Single-cell RNAseq assesses the transcriptome stage-specific signature of LRR5*pB*

**Fig 1:**
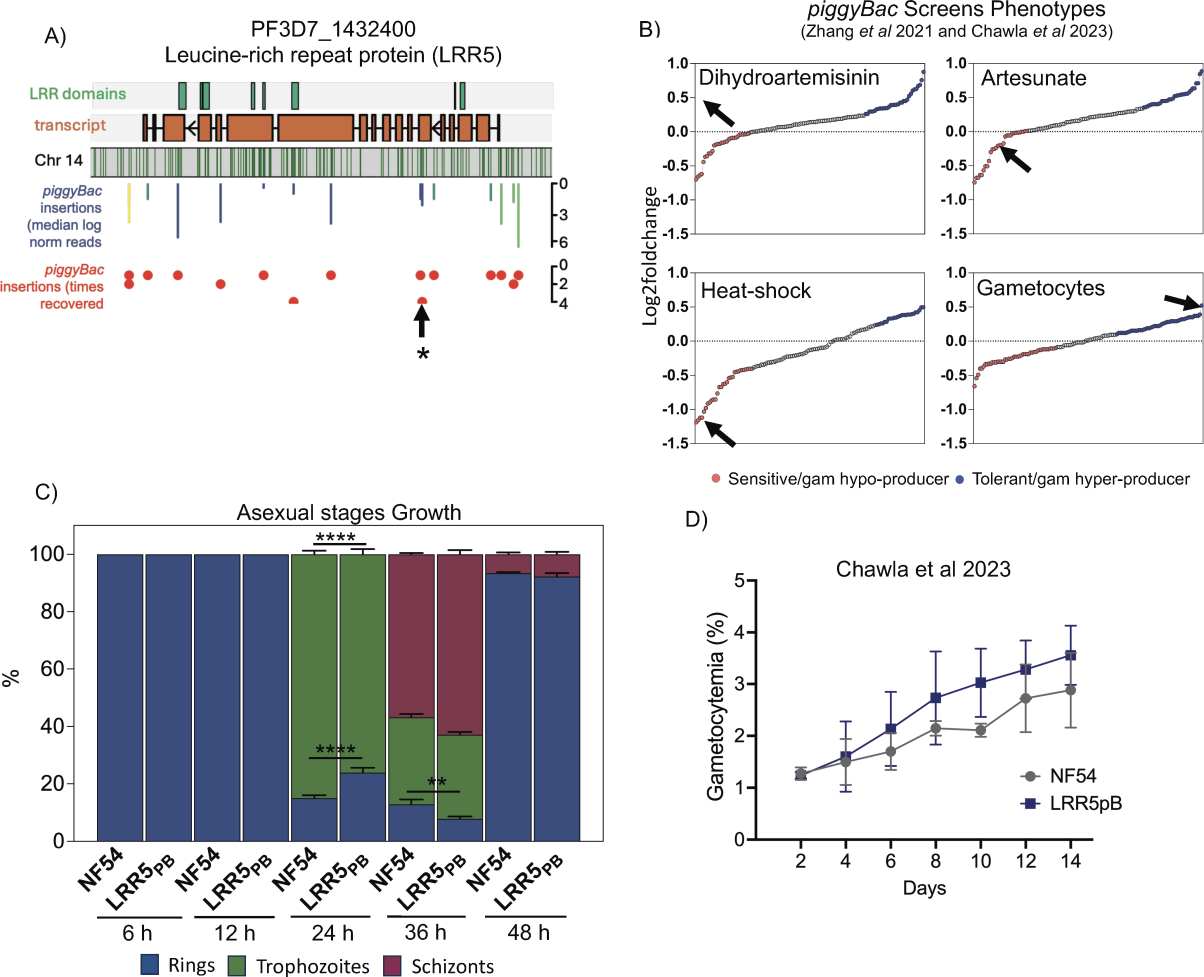
Phenotypic characterization of the LRR5pB mutant. (**A**) Schematic representation of the *LRR5* gene (*PF3D7_1432400*) based on previously published *piggyBac* saturation mutagenesis data (12). *LRR5* gene comprises 19 exons and encodes a protein containing specify number leucine-rich repeat (LRR) domains. Multiple *piggyBac* insertions recovered across the coding region classify *LRR5* as dispensable for asexual blood-stage growth. The arrow indicates the specific *piggyBac* insertion site in exon 6 of the cloned LRR5pB mutant used in this study. (**B**) Previously reported phenotypes of the LRR5pB mutant, including increased sensitivity to dihydroartemisinin (DHA)**,** artesunate**,** and febrile (heat-shock) conditions (17), as well as classification as a gametocyte hyperproducer in genome-wide gametocytogenesis screens (16). (**C**) Proportions of asexual blood-stage parasites in LRR5pB and NF54 wild-type lines. Highly synchronized ring-stage cultures (0–6 h post-invasion [hpi]) were initiated at 0.25% parasitemia and monitored over a 48-h intraerythrocytic cycle. Giemsa-stained smears were collected at ~6, 12, 24, 36, and 48 hpi. Parasitemia and the percentages of ring, trophozoite, and schizont stages were determined by counting 10,000 red blood cells per replicate. Data represent three biological replicates**,** shown as means ± standard deviation (SD). Comparisons among parasite lines in each stage were analyzed using two-way ANOVA, followed by Šídák’s multiple comparisons test (*P < 0.01, **P < 0.001, ***P < 0.0001, ****P < 0.00001). (**D**) Gametocyte development of LRR5pB and NF54 parasites over 14 days extracted from reference 16. The LRR5pB mutant consistently produced higher gametocyte numbers than wild-type parasites at all time points, confirming its gametocytes hyper-producer phenotype (16).

LRR5 is a conserved *P. falciparum* leucine-rich repeat protein (1,504 amino acids) characterized by multiple LRR motifs within its domain architecture. ws*PiggyBac* mutagenesis profiling identified LRR5 as dispensable for asexual blood-stage growth under ideal *in vitro* culture conditions (Mutagenesis Index Score = 1), with multiple (≥15) independent insertions detected across its exons, indicating tolerance to disruption. The specific mutant analyzed in this study, LRR5pB, carries a *piggyBac* insertion within exon 6 (PF3D7_14_m1::1281184) (Fig. 1A). Previous chemogenomic and stress-response screens revealed that LRR5pB exhibits increased sensitivity to artemisinin derivatives—including dihydroartemisinin (DHA) and artesunate—as well as to febrile (heat-shock) conditions (17) (Fig. 1B). Interestingly, in a screen for mutants with alterations in gametocyte production, the same mutant was identified as a significant hyper-producer of gametocytes (16)(Fig. 1B). We grew tightly synchronized cultures of LRR5pB in parallel with the isogenic wild-type (WT) NF54 line and quantitated developmental stages using Giemsa staining and light microscopy (Fig. 1C). No major morphological defects were observed although there was a statistically significant reduction in the abundance of trophozoite-stage parasites in LRR5pB at 24 h (*P* < 0.00001) and an increased abundance of schizonts at 36 h (*P* < 0.001), suggesting alterations in development. Previous data (Fig. 1D, from reference 16) showed that this mutant had an increased rate of gametocyte production. These changes in development in both asexual and sexual stages suggest that LRR5 disruption subtly alters regulatory pathways involved in growth and development.

**Fig 2:**
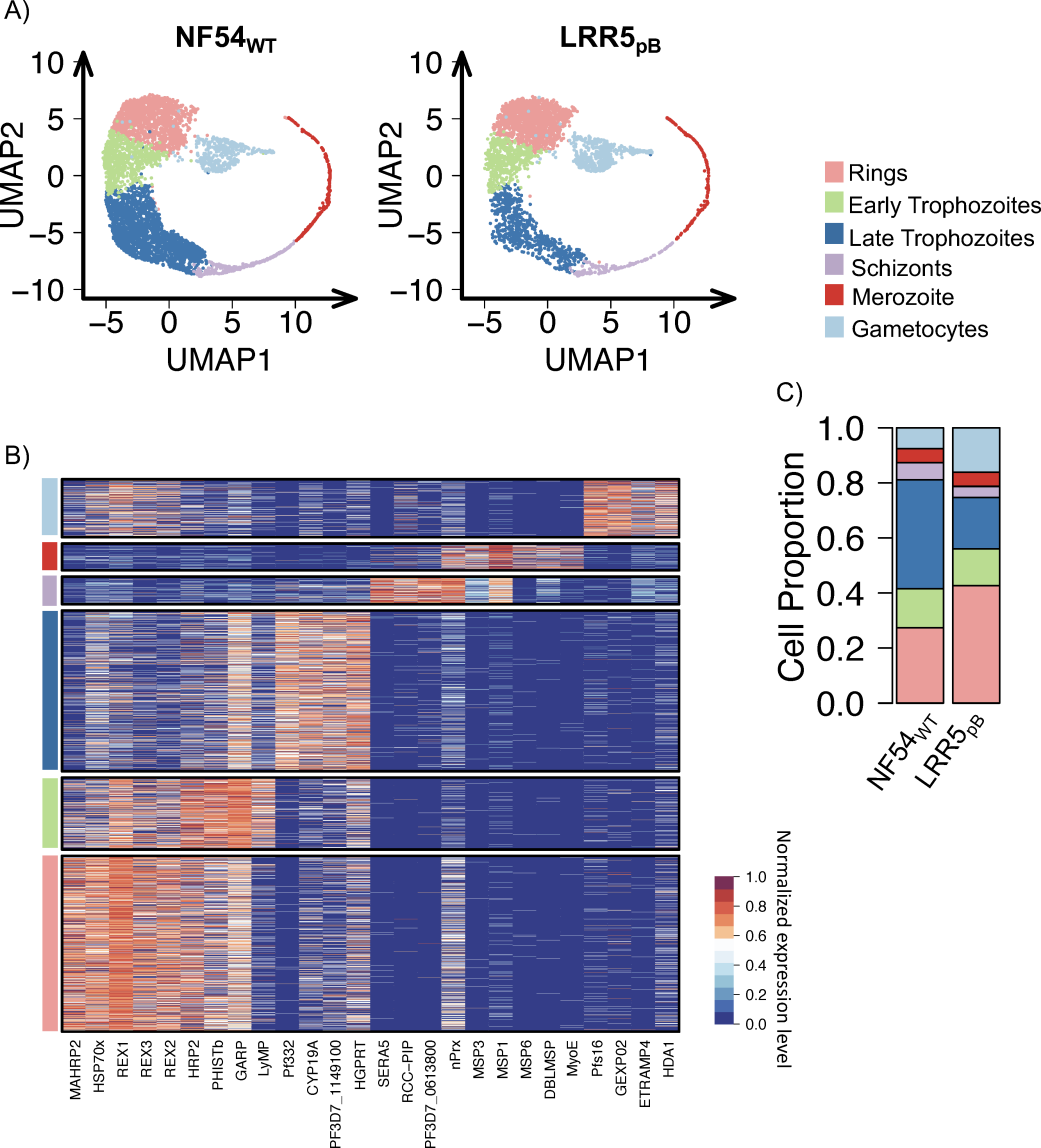
Single-cell transcriptomic profiling identifies developmental stage-specific clusters in LRR5pB and NF54 parasites. (**A**) Uniform Manifold Approximation and Projection (UMAP) visualization of single-cell RNA sequencing data from LRR5pB and NF54 wild-type parasites, with cells colored according to their assigned intraerythrocytic developmental stages. (**B**) Normalized expression levels of stage-specific marker genes used to annotate the life cycle stages within each transcriptional cell cluster. (**C**) Relative proportions of transcriptional clusters of LRR5pB and NF54_WT_ parasite populations.

To characterize the transcriptomic impact of LRR5 disruption across parasite developmental stages, we performed single-cell RNA sequencing (scRNA-seq) using the 10× Genomics Chromium platform on LRR5pB and NF54 wild-type blood stage parasites. To capture the cellular heterogeneity and developmental complexity of *P. falciparum* within human RBC infections, we leveraged the resolving power of scRNA-seq and optimized our workflow to analyze unsynchronized parasite populations without prior enrichment procedures (e.g., Percoll gradients or magnetic isolation) in contrast to previous *Plasmodium* single-cell studies (25–28). This approach generated a data set of 8,290 single cells in both parasite lines (4,835 NF54 wild-type and 3,455 LRR5pB mutant) which was then projected on UMAP (Uniform Manifold Approximation and Projection), displaying a semi-circular arrangement with branching cells consistent with other malaria single-cell RNAseq studies (29–31). This analysis identified seven transcriptionally distinct clusters (Fig. 2B) based upon the expression of validated stage-specific marker genes (Fig. 2B; Data Set S1). These clusters captured the expected continuum of the *P. falciparum* intraerythrocytic cycle, including ring, trophozoite (early and late), schizont, and merozoites stages, as well as gametocytes (Fig. 2B and C) (Table S1). Notably, although parasites were maintained under asexual blood-stage culture conditions without deliberate gametocyte induction, a distinct gametocyte transcriptional cluster, expressing canonical gametocyte marker genes such as GEXP02 (PF3D7_1102500, gametocyte exported protein 2) and Pfs16 (PF3D7_0406200, parasitophorous vacuole membrane protein S16), was identified in both WT and LLR5pB lines.

Quantifying the number of cells within each developmental cluster revealed an increased representation of cells expressing ring-stage genes in the LLR5pB line (Fig. 2C) consistent with morphological observations (Fig. 1C), where LRR5pB parasites exhibited a prolonged ring-stage profile. Consistent with its previously reported hyper-gametocyte producer phenotype (16) the other observed changes (Fig. 1B through D), the gametocyte-associated cluster was also expanded in LRR5pB compared with NF54 (Fig. 2C), suggesting that LRR5 disruption increases the proportion of parasites transcriptionally committed to gametocytogenesis.

### LRR5 disruption dysregulates protein-folding and host-interaction pathways while promoting early gametocyte gene expression

**Fig 3:**
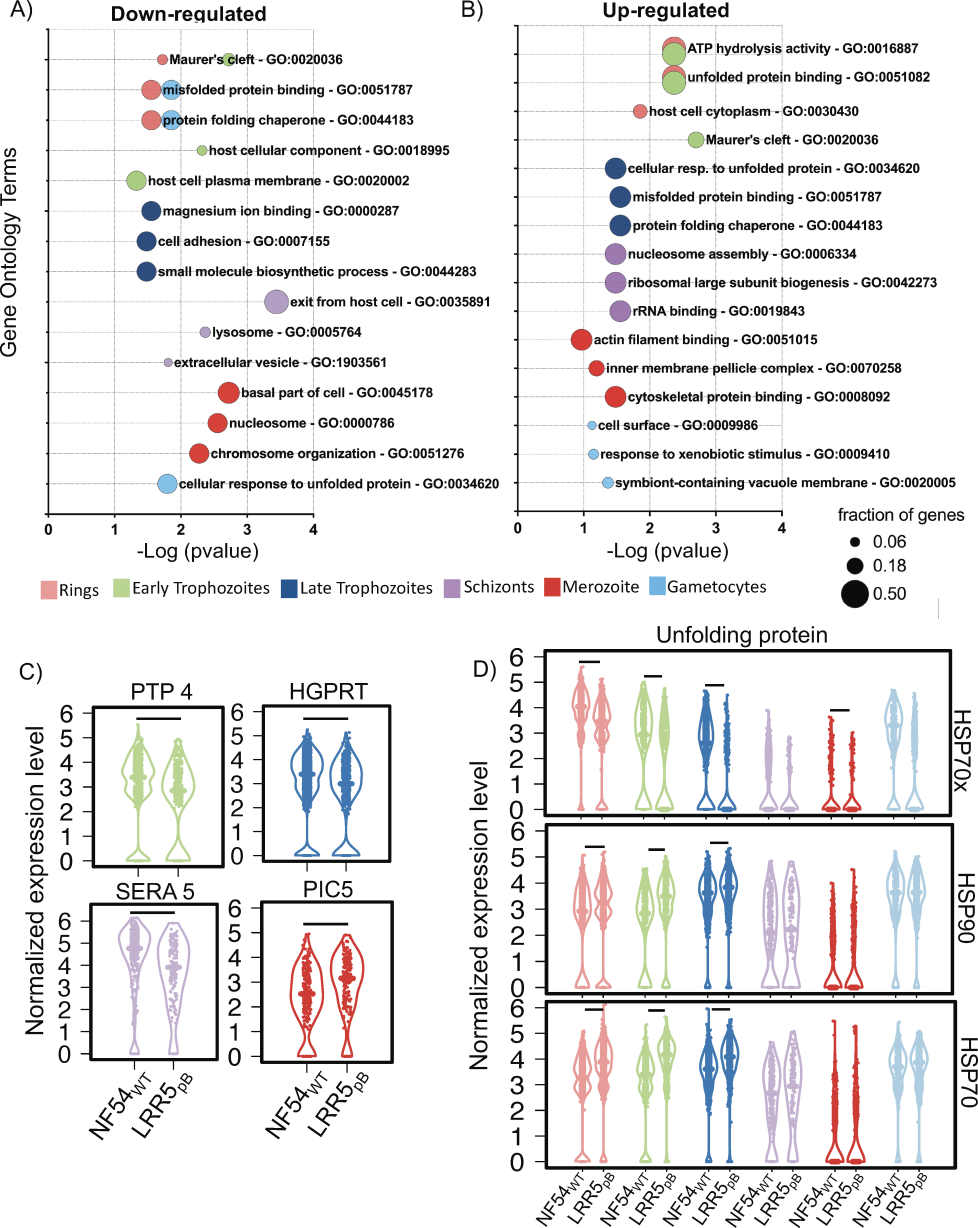
LRR5 disruption alters stress-response and host-interaction transcriptional programs. (**A and B**) Gene Ontology (GO) enrichment analysis of differentially expressed genes comparing LRR5pB and NF54 wild-type parasites. Shown are the most significantly downregulated (**A**) and upregulated (**B**) biological process terms in LRR5pB. (**C**) Expression levels (log of reads per million RPM) of representative downregulated genes in LRR5pB across developmental stages, including PTE4 (PF3D7_0730900; EMP1-trafficking protein) in early trophozoites, HGPRT (PF3D7_1012400; hypoxanthine–guanine phosphoribosyltransferase) in late trophozoites, SERA5 (PF3D7_0207600) in schizonts, and PIC5 (PF3D7_1310700; PhIL1-interacting candidate) in merozoites. Black bars above plots indicate statistically significant differential expression (Wilcoxon rank-sum test). (**D**) Expression levels (RPM) of heat-shock protein chaperones across the parasite life cycle, including HSP70-x (PF3D7_0831700), HSP90 (PF3D7_0708400), and HSP70 (PF3D7_0818900). Black bars above plots indicate statistically significant differential expression (Wilcoxon rank-sum test).

Differentially expressed genes (DEGs) were identified between NF54 and LRR5pB parasites, followed by Gene Ontology (GO) enrichment analysis to determine the biological processes affected by LRR5 disruption (Fig. 3 and Tables S2 and S3), analyzed within each transcriptional cluster. In total, 42 genes were upregulated in LRR5pB across all developmental stages (rings, *n* = 10; early trophozoites, *n* = 4; late trophozoites, *n* = 10; schizonts, *n* = 3; merozoites, *n* = 12; and gametocytes, *n* = 3), while 17 genes were downregulated (rings, *n* = 3; early trophozoites, *n* = 4; late trophozoites, *n* = 12; schizonts, *n* = 3; merozoites, *n* = 4; and gametocytes, *n* = 1). Differential expression was defined using thresholds of |log_2_FC| >= 0.25 and adjusted *P* value (padj) < 0.01 (Table S2). The GO analysis revealed an altered transcriptional landscape for the LRR5pB parasite characterized by dysregulation of genes involved in host–parasite interaction, protein trafficking, and protein folding. Among the most significantly downregulated pathways were those associated with Maurer’s cleft organization, host-cell plasma membrane remodeling, cell adhesion, and extracellular structure organization (GO:0044409, GO:0031226, GO:0007155, and GO:0043062) (Fig. 3A and B). Transcripts for genes linked to these pathways were consistently reduced across multiple stages of the asexual cycle—from rings through trophozoites, schizonts, and merozoites, indicating that LRR5 disruption compromises the parasite ability to remodel and communicate with its host erythrocyte. Transcripts from genes encoding several exported proteins critical for host-cell modification were among the most downregulated, including PTE4 (PF3D7_0730900, EMP1-trafficking protein), a Maurer’s cleft-associated trafficking factor; SERA5 (PF3D7_0207600), a parasitophorous vacuole-localized protease; and PIC5 (PF3D7_1310700), a PhIL1-interacting component of the inner membrane complex (Fig. 3C). These factors play critical roles in protein export and host-cell remodeling, suggesting that LRR5pB parasites have impaired virulence-associated trafficking functions.

Another group of downregulated genes were those related to protein folding and chaperone activity (GO:0051787, GO:0044183), particularly within the ring-stage and gametocyte clusters (Fig. 3A). Notably, the HSP70-x chaperone (PF3D7_0831700) was significantly downregulated in LRR5pB (Fig. 3D). HSP70-x is a heat-shock protein exported to the host erythrocyte, where it facilitates the folding and trafficking of parasite effectors and supports stress tolerance and cytoadherence (32–35). Its downregulation, together with decreased transcript levels of host-cell remodeling genes, suggests a weakened proteostasis network in the mutant—processes especially important in the unfolded protein response. Interestingly, LRR5pB also showed the upregulation of cytoplasmic HSPs (PF3D7_0708400, HSP90, heat shock protein 90 and PF3D7_0818900, HSP70, heat shock protein 70) and translation-related pathways, possibly reflecting a compensatory response. Genes involved in ribosome biogenesis and translation initiation (GO:0011843, GO:0042273) were elevated in trophozoite stages (Fig. 3A), while those associated with membrane or surface remodeling were enriched in merozoite and gametocyte clusters (Fig. 3B). This shift suggests that LRR5pB parasites attempt to offset folding defects by enhancing global protein synthesis and stress-response capacity.

**Fig 4:**
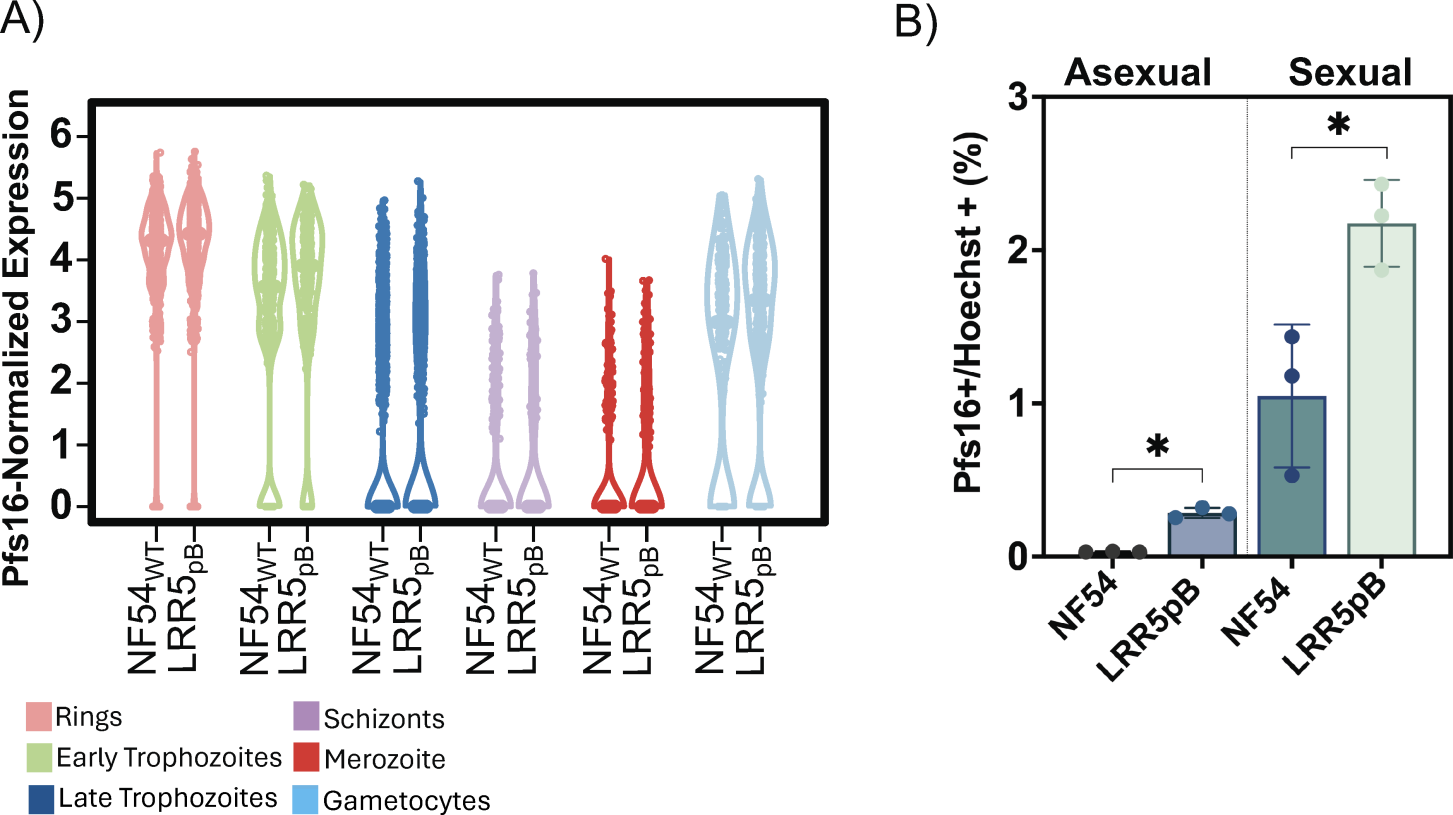
Premature activation of the early gametocyte marker Pfs16 in LRR5pB parasites. (**A**) Expression levels (reads per million, RPM) of Pfs16 (PF3D7_0406200; parasitophorous vacuole membrane protein S16) within the gametocyte cluster, showing significant elevated Pfs16 transcript abundance in LRR5pB compared with NF54 wild-type parasites in the gametocyte cluster (Table S2). (**B**) Flow cytometric quantification of Pfs16 protein expression in NF54 wild-type and LRR5pB parasites stained with anti-Pfs16 antibodies under asexual (non-induced) and sexual (gametocyte-induced) culture conditions. Bars represent the mean fluorescence intensity (MFI) of Pfs16^+^/Hoechst^+^ parasites from three independent biological replicates, with error bars indicating standard deviation. LRR5pB parasites exhibit significantly higher Pfs16 protein expression than NF54 under both asexual and sexual conditions (*P < 0.01, Welch’s *t* test; see Materials and Methods).

A key finding was the upregulation of the early gametocyte marker Pfs16 in LRR5pB, even under asexual culture conditions without gametocyte induction. Pfs16, a major component of the parasitophorous vacuole membrane during early gametocytogenesis (36–39), showed elevated transcript levels in LRR5pB (Fig. 4A), suggesting premature activation of sexual commitment. This transcriptional pattern was confirmed at the protein level by flow cytometry using anti-Pfs16 antibodies, which revealed that LRR5pB parasites express Pfs16 at significantly increased levels compared with NF54 PFs16 expression (Fig. 4B). These data indicate that LRR5 disruption shifts the early intraerythrocytic developmental processes toward the activation of gametocyte commitment pathways, supporting the gametocytes hyper-producer phenotype observed for this mutant (16).

Together, these results define a distinct transcriptional signature for the LRR5pB line that is characterized by impaired host-cell remodeling, altered protein-folding, and premature activation of translational and gametocytogenesis gene programs (Fig. 3 and 4). This imbalance likely underlies the mutant’s heightened sensitivity to heat shock and artemisinin-induced stress (Fig. 1B) and highlights a potential association between LRR5 and parasite stress responses, from protein unfolding and host remodeling to developmental signaling and timing.

### Pseudotime analysis reveals premature activation of trophozoite, merozoite, and gametocyte gene clusters in LRR5pB

Differential expression analysis indicated that the disruption of LRR5 leads to transcriptional dysregulation across pathways related to protein unfolding, translation, host-parasite interaction, and gametocyte development. Given this and the increased proportion of ring- and gametocyte-enriched clusters in the mutant (Fig. 2), we applied Monocle 3-based pseudotime trajectory analysis to examine how LRR5 disruption influences transcriptional progression throughout the intraerythrocytic developmental cycle. Because direct temporal tracking of individual parasites is not possible, pseudotime analysis was used to order single cells according to transcriptional similarity, providing a proxy for developmental progression.

**Fig 5:**
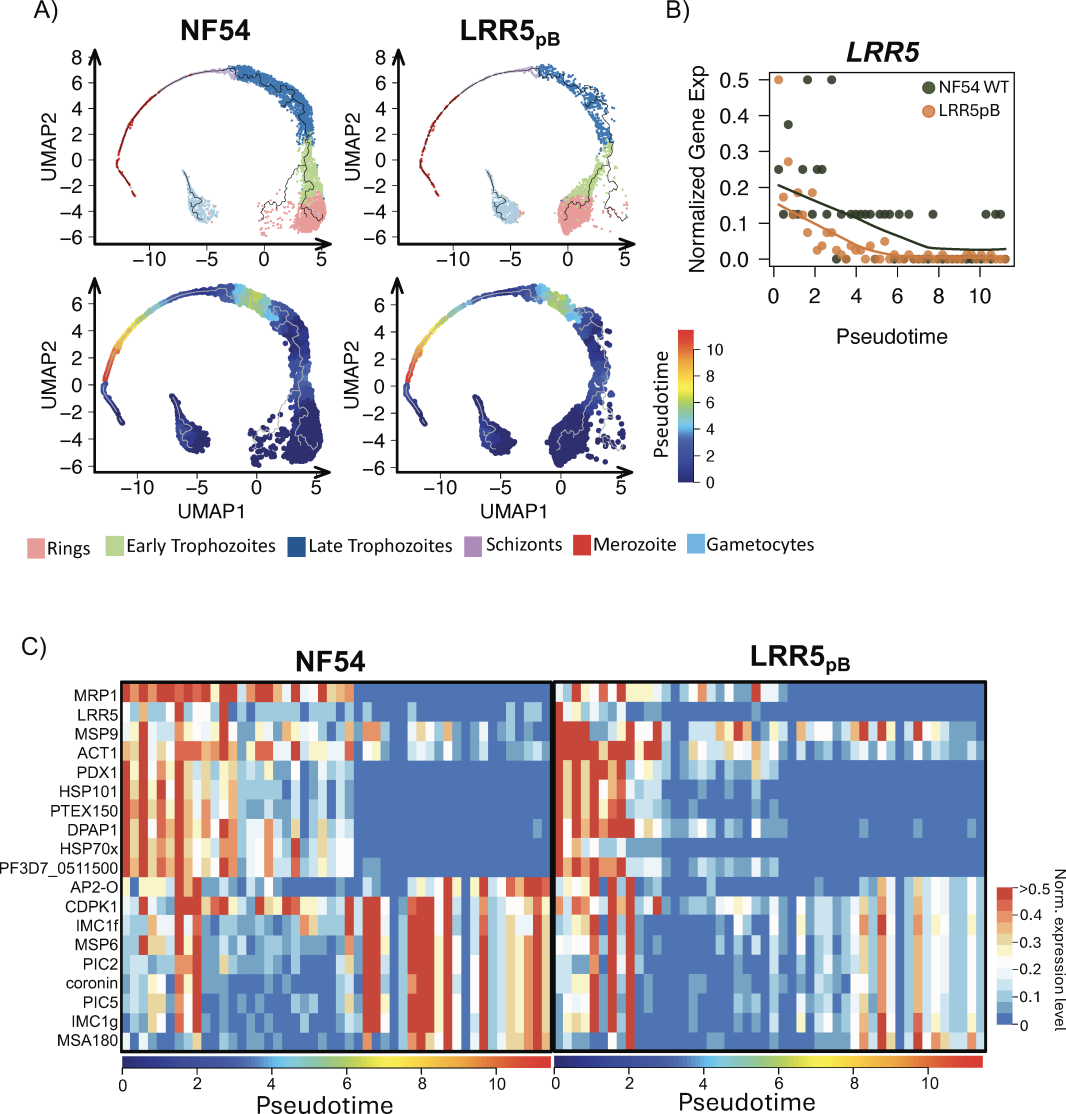
Pseudotime trajectory analysis of transcriptional timing in LRR5pB and NF54 parasites. (**A**) Pseudotime trajectories inferred by Monocle 3 overlaid onto the UMAP of NF54 and LRR5pB parasites. The reconstructed trajectory captures the expected developmental progression across the intraerythrocytic cycle. Cells are colored by annotated intraerythrocytic developmental stages (above panel) colored according to pseudotime progression (blue to red), representing inferred developmental advancement based on transcriptional similarity (below panel). (**B**) Pseudotime distribution of cells from NF54 and LRR5pB parasites. Each dot represents an individual single cell, positioned according to its inferred pseudotime value. The solid lines represent the fitted trajectory (smoothed trend) of gene-expression progression across pseudotime for each parasite line. Comparison of the distributions indicates that LRR5pB cells exhibit a leftward shift along the trajectory, consistent with earlier activation of stage-associated transcriptional programs relative to NF54. (**C**) Heatmap of selected significantly mistimed genes (*q* < 0.05) ordered along pseudotime, illustrating altered transcriptional timing in LRR5pB relative to NF54. Gene expression values are shown after normalization as described in the Methods. The entire list of significantly mistimed genes (*q* < 0.05) is provided in Table S4.

The pseudotime reconstruction (Fig. 5A) identified a continuous developmental trajectory spanning early to late asexual stages. When LRR5pB and NF54 parasites were compared along this trajectory, *LRR5* gene shows significant earlier expression (*q* < 0.05) in the LRR5pB than in the NF54 wild-type line (Fig. 5B). This shift indicates that LRR5 disruption perturbs the temporal coordination of 61 genes expression programs, rather than altering stage identity itself. Heatmap visualization of 19 significant mistimed (*q* < 0.05) pseudotime-ordered genes (Fig. 5C; Table S4) revealed premature expression on LRR5pB of components of the PTEX protein-export complex (HSP101, EXP1) and HSP70-x, as well as inner membrane complex (IMC) proteins such as IMC1f (PF3D7_1351700), IMC1g (PF3D7_0525800), PIC2 (PF3D7_0822900), and PIC5 (PF3D7_1310700), displayed earlier or mistimed expression relative to NF54. Similarly, the calcium-dependent protein kinase 1 (CDPK1, PF3D7_0217500), a regulator of IMC phosphorylation during schizogony (40, 41), was also expressed earlier in LRR5pB. These results indicate that LRR5 disruption leads to premature activation of genes involved in protein export, IMC organization, and merozoite maturation (MSA180, PF3D7_1014100; and MSP9, PF3D7_1228600)—potentially altering cell-cycle coordination and developmental timing.

Taken together, the differential expression/GO analysis and the pseudotime trajectory analysis provide complementary but distinct insights into the transcriptional consequences of LRR5 disruption. The differential expression genes and gene ontology analysis identify which pathways are altered in magnitude between LRR5pB and NF54 within defined developmental clusters, highlighting reduced expression of genes involved in host-cell remodeling, protein export, and protein folding, and compensatory upregulation of translation and cytoplasmic stress-response pathways. The pseudotime analysis demonstrates how the altered timing of expression of a subset of genes in similar pathways is altered along the developmental trajectory but does not reveal additional pathways. Notably, several genes identified as downregulated or dysregulated in the GO analysis, such as HSP70-X, PTEX components, and IMC proteins including PIC5, also appear among the mistimed genes in pseudotime, where they are activated earlier in LRR5pB relative to NF54.

Although no measurable difference in overall asexual growth rate was observed between LRR5pB and NF54 (Fig. 1), the upregulation of translational and structural genes, together with mistimed activation of export and IMC components, likely contributes to the precocious expression of the early gametocyte marker Pfs16. This early activation of gametocyte commitment pathways is consistent with the gametocyte hyper-producer phenotype previously described for the LRR5pB mutant (16) and is consistent with a multi-faceted impact of LRR5 disruption on the timing of stress adaptation and developmental transitions.

## DISCUSSION

The in-depth transcriptional analysis of this study revealed how LRR5 disruption reshapes the *Plasmodium falciparum* transcriptional landscape, linking stress responses, protein-folding networks, and host–parasite interactions with the premature activation of gametocyte-associated genes. The single-cell transcriptomics resolved subtle, stage-specific transcriptional differences consistent with altered stress responses and developmental timing in malaria parasite, are consistent with the experimentally defined phenotypes of the mutant, including high sensitivity to heat shock and artemisinin and increased gametocyte production.

Our findings suggest that LRR5 plays a role in signaling in processes linking environmental stress responses with developmental progression. Leucine-rich repeat (LRR) proteins are well known for mediating protein–protein interactions (21, 42), and their key roles in stress sensing and innate immunity are well established in plants (42–44), mammals (44–46), and insects. Specifically, an LRR-containing protein complex activates mosquito complement as a defense mechanism against *P. berghei* parasites (47, 48). In *Plasmodium,* LRR proteins are best characterized in mosquito interactions: in *P. berghei,* the LRR protein PbLRR1 downregulates protein phosphatase 1 (PP1) activity and is required for efficient oocyst development (22), while in *P. falciparum*, LRR1 binds to serine/threonine phosphatase type 1 and inhibits parasite growth *in vitro* (23). *P. falciparum* encodes 14 LRR-containing proteins, yet their roles in blood-stage development remain largely unknown. The broad transcriptional adjustments observed following LRR5 disruption—including altered expression of heat-shock, protein-export, and signaling genes—support the hypothesis that LRR5 participates in stress-sensing pathways that fine-tunes gene expression for proteostasis and developmental timing. Moreover, the premature activation of gametocyte-associated transcripts in the LRR5pB mutant (Pfs16) suggests that LRR5 perturbation is associated with disrupted synchrony between stress adaptation and stage progression.

The mistimed expression of CDPK1 and IMC-related genes in LRR5pB indicates that this disruption affects calcium-dependent signaling pathways essential for membrane remodeling. CDPK1 is a central regulator of schizogony and merozoite egress, controlling phosphorylation of IMC components (41, 49–51) and activating proteases such as SERA5 (40), whose phosphorylation by CDPK1 is required for its proteolytic activity and egress (40). Consistent with this, transcripts of SERA5 and PIC5 were altered in LRR5pB, suggesting that LRR5 disruption is associated with altered expression of genes converging on the IMC and egress machinery. The LRR architecture (21, 24, 45) of LRR5 likely facilitates protein–protein interactions, as observed in mosquito and *P. berghei* (47) that modulate these calcium-driven events. Under stress conditions such as heat shock or artemisinin exposure, aberrant LRR5 function may trigger premature remodeling or export processes, resulting in impaired merozoite development. These molecular alterations align with the phenotypic sensitivity of LRR5pB parasites to febrile and drug stress (17) and the slight perturbations observed during asexual-stage progression in standard culture conditions (Fig. 1), suggesting that LRR5 contributes to maintaining the balance between survival and replication efficiency.

The mistimed and down-expression of exported chaperones, specifically HSP70-x, that is essential for parasite virulence (32) and trafficking components of the PTEX complex (PTEX150 and HSP101), further indicates that LRR5 disruption compromises both proteostasis and host-cell remodeling. The PTEX machinery exports hundreds of parasite proteins across the parasitophorous vacuole membrane (PVM), ensuring proper remodeling of the infected erythrocyte (35, 52–55). HSP70-x is secreted into the parasitophorous vacuole, where it interacts with PTEX (32, 34) and is also exported into the host erythrocyte (55, 56). Altered timing or reduced expression of PTEX components and HSP70-x suggests a disturbance in protein trafficking and refolding within the host cell, likely impairing the parasite’s capacity to manage febrile or drug-induced protein damage. Interestingly, increased expression of other cytoplasmic HSPs (HSP70, HSP90; Fig. 3) may partially compensate for reduced HSP70-x activity, preserving limited folding capacity. Given that LRR proteins often mediate protein–protein interactions, LRR5 may be associated with stress-responsive export and signaling pathways, indirectly influencing PTEX and IMC function under stress. These combined effects likely explain the heightened sensitivity of LRR5pB parasites to DHA and heat shock, as well as their slower growth relative to the parental NF54 line.

A distinctive feature of LRR5pB is the premature and enhanced expression of Pfs16, an early gametocyte marker localized to the PVM (36, 57–59). Validation of Pfs16 protein upregulation supports the transcriptional data, indicating that a greater proportion of parasites are transcriptionally primed for gametocytogenesis. This shift may result from altered calcium signaling or stress-responsive pathways involving CDPK1, HSP70-x, and PTEX-associated remodeling, which are known to influence membrane stability and vacuolar trafficking. The convergence of these perturbed systems suggests that LRR5 plays a role in the inner-membrane formation and vacuolar processes required for both egress and early gametocyte formation (57, 60–63). Thus, the transcriptional signatures of stress adaptation, protein export, and gametocyte activation observed in LRR5pB likely reflect a dysregulated regulatory network that couples proteostasis to developmental commitment.

We propose that LRR5 plays an important role in processes that balance stress resilience and developmental plasticity. In wild-type parasites, LRR5-dependent signaling promotes proteostasis and host-cell remodeling, enabling recovery under stress. When LRR5 is disrupted, this adaptive coordination breaks down, predisposing parasites to alternative survival strategies such as gametocytogenesis. The LRR5pB phenotypes, characterized by increased sensitivity to stress and enhanced gametocyte production, are consistent with previous observations that conditions reducing asexual viability, including oxidative or proteotoxic stress, can trigger sexual conversion as a persistence mechanism (16, 17).

In conclusion, our study demonstrates how scRNA-seq can resolve the complex interplay between stress responses and developmental regulation in malaria parasites. The robustness of these findings is reinforced by reproducible phenotypes, protein expression validation, and agreement with independent data sets, demonstrating consistency despite limited scRNA-seq sampling. By capturing transcriptional heterogeneity across the asexual-to-sexual, our data reveal how LRR5 disruption reshapes temporal gene-expression patterns and exposes hidden associations between proteostasis, export, and differentiation signaling. Future studies will aim to identify LRR5-interacting partners and determine whether the LRR domain can directly mediate stress sensing or signal transduction via kinase or calcium networks. Such insights may clarify how *P. falciparum* balances survival and transmission under environmental or therapeutic stress, providing new leads for interventions targeting gametocyte formation.

## MATERIALS AND METHODS

### Parasite culture and maintenance

The *P. falciparum* parasite cultures (*piggyBac* mutant and NF54 wild-type) were cultured at 4% hematocrit (O^+^ erythrocytes) (https://bioivt.com/) in complete media containing RPMI 1640 (KD Medical), 2.5% sodium bicarbonate (prepared from a 7.5% stock), supplemented with 50 µg/mL hypoxanthine and 25 mM HEPES. Flasks were incubated at 37°C with a continuous gas mixture of 90% N₂, 5% CO₂, and 5% O₂.

### Generation of *piggyBac* mutant clone

Isogenic *piggyBac* insertion mutants were first created by transposon-mediated mutagenesis and validated by quantitative insertion site sequencing (QIseq) (13, 64–66). For this study, the LRR5 *piggyBac* mutant clone was retrieved from cryostorage, thawed, and returned to culture; after recovery, it was maintained in a T25 flask until parasitemia reached ~2% and then expanded as needed for downstream assays.

### Intraerythrocytic developmental cycle and growth analysis

The *P. falciparum* LRR5 *piggyBac* (LRR5pB) mutant was evaluated for alterations in intraerythrocytic developmental cycle progression compared to the isogenic wild-type NF54 clone. Parasite stages were monitored over a complete 48-h cycle by microscopy, quantifying the proportions of ring, trophozoite, and schizont forms. Both parasite lines were first synchronized at the schizont stage using Percoll density gradients and cultured in 4% hemtocrit, 37°C, under a continuous gas flow of 90% N₂, 5% CO₂, and 5% O₂. When the proportion of ring stages exceeded 0.5%, cultures were treated with sorbitol and adjusted to 1% parasitemia at 4% hematocrit. Culture media was replaced and Giemsa-stained thin smears were prepared from NF54 and LRR5pB cultures every 6 h for 48 h. Parasitemia and stage distributions were calculated by counting at least 10,000 RBC per sample (per slide), expressing each developmental stage as a percentage of total parasites. All assays were performed in triplicate using independent biological replicates.

### Parasite culture for single-cell RNA-sequencing library preparation

Parasitemia of asynchronous NF54 and LRR5pB parasite culture were determined by counting infected iRBCs on Giemsa-stained smears. Total RBC concentrations were obtained using hemocytometer counting. Parasitemia values (iRBCs/µL) were used to normalize sample input.

For single-cell capture, infected red blood cells (iRBCs) were washed and resuspended in PBS containing 0.04% BSA. Approximately 10,000 iRBCs per sample were loaded onto a 10× Genomics Chromium Controller for GEM generation and barcoding using the Single Cell 3′ Gene Expression chemistry. Reverse transcription was performed within GEMs, followed by cDNA purification and amplification. Library concentrations were quantified with a Qubit fluorometer, and fragment size distributions were assessed using a Bioanalyzer or Fragment Analyzer. Amplified cDNA was then processed to generate Illumina short-read 10 × 3′ gene expression libraries following the manufacturer’s protocol, including enzymatic fragmentation, end-repair/A-tailing, adaptor ligation, and sample indexing. Indexed libraries were pooled and sequenced on an Illumina NextSeq 2000, with Read 1 capturing cell barcodes and UMIs and ~90 bp of cDNA sequence captured in Read 2.

### Single cell RNA-seq analysis

The original reads from LRR5pB and NF54WT were mapped to the *P. falciparum* genome (11) (PlasmoDB v68 [10]) using Cell Ranger (67) with default parameters. Doublets were identified and removed from each data set (LRR5pB and NF54WT) using Scrublet (68) with a doublet score cutoff of 0.4. The remaining cells were further filtered to retain those expressing between 200 and 1,500 genes, with more than 500 total counts per cell, and less than 35% mitochondrial gene content. Normalization was performed using the SCTransform function in the Seurat package (v5.3.0) (69) with method = "glmGamPoi" and vars.to.regress = "percent.mt". Integration of the LRR5pB and NF54WT data sets was conducted using SelectIntegrationFeatures and FindIntegrationAnchors with 2,000 selected features and SCT normalization. The final integration was performed using IntegrateData (70). Principal component analysis (PCA) was carried out, and the top 20 dimensions were used to generate the UMAP. Marker genes for each UMAP-defined cell cluster were identified using log-normalized expression profiles with the FindAllMarkers function. Genes were considered cluster markers if they exhibited an absolute log₂ fold change greater than 0.264 (corresponding to a fold change >1.2) compared with other cell clusters, p-adjustment lower than 0.01, and were expressed in at least 75% of cells within the cluster. Differentially expressed genes between LRR5pB and NF54WT across all clusters were identified using the FindMarkers function, applying the same thresholds of absolute log₂ fold change >0.264 and a minimum expression in 75% of cells (Tables S1 and S2).

Cellular trajectory (Pseudotime) (71) was estimated using Monocle 3 (v1.4.26) with Seurat-processed integrated objects as input. Dimensionality reduction was performed with the reduce_dimension function using 50 principal components. Cells were clustered using cluster_cells with 10 nearest neighbors. The trajectory graph was learned with learn_graph, and pseudotime was inferred by ordering cells (order_cells) with root cells corresponding to the ring-stage population. Final pseudotime values were obtained using the pseudotime function. Gene expression dynamics were modeled as a function of both experimental condition (LRR5pB or NF54WT) and pseudotime using Monocle 3. For each gene, we fit a model of the form Expression ~Status + splines::ns(pseudotime, df = 1) + Status × splines::ns(pseudotime, df = 1), where Status represents the sample group, and ns(pseudotime, df = 1) specifies a natural spline basis that flexibly captures nonlinear expression trends along pseudotime. The interaction term (Status × splines::ns (pseudotime, df = 1)) was used to assess whether gene expression trajectories differ between LRR5pB and NF54WT across the inferred pseudotime. Genes showing significant interaction effects (*q* < 0.05) were considered differentially expressed through pseudotime between the two conditions (Table S4).

### Gene ontology enrichment analysis

GO-enrichment analyses were performed by testing GO-terms mapped to the phenotypic categories of interest against a background of GO-terms mapped to all other genes using our R package pfGO (72) (v 1.1). Categories of interest were downregulated in rings (down-rings), early-trophozoites (down-ET), late-trophozoites (down-LT), Schizonts (down-schiz), merozoites (down-Mer), and gametocytes (down-gam); upregulated in downregulated in rings (up-rings), early-trophozoites (up-ET), late-trophozoites (up-LT), Schizonts (up-schiz), merozoites (up-Mer), and gametocytes (up-gam); and neutral in rings (none-rings), early-trophozoites (none-ET), late-trophozoites (none-LT), Schizonts (none-schiz), merozoites (none-Mer), and gametocytes (none-gam) (Fig. 3). The GO-term database was created from the latest curated *P. falciparum* ontology available at the time of analysis from PlasmoDB (73), and enrichment was assessed via a weighted Fisher/elim-hybrid *P* <=0.05 (v. 57). The fraction of genes represents the number of significant genes annotated to a given GO-term in each of the categories divided by the total number of genes annotated to that GO-term included in the analysis for all categories (background-set). The entire GO-data set is provided in Table S3.

### *P. falciparum* gametocyte induction and Pfs16 flow cytometer counts

NF54 wild-type and the LRR5 *piggyBac* mutant were induced to gametocytogenesis in parallel using the standard “crash” induction with minor adjustments (74, 75). Briefly, asexual cultures were expanded to ~4% parasitemia under routine conditions. Culture was grown >7% total parasitemia with approximately 80% rings. To initiate gametocytogenesis, cultures were “crashed” by reducing parasitemia to 1% without adding new red blood cells (i.e., no increase in hematocrit). Three biological replicates were used for phenotypic characterization. Cultures were then maintained with complete medium changes once per day for 4 days post-induction, without adding fresh erythrocytes.

Then, 200 µL aliquots of gametocyte cultures were transferred to 96-well and pelleted with two technical replicates for each sample. Pellets were washed three times with incomplete RPMI and then blocked with 200 µL of 3% BSA in PBS for 30 min at room temperature (RT). After centrifugation and removal of the blocking solution, cells were washed three times with PBS. For primary staining, pellets were resuspended in FACS buffer (PBS, 1% BSA) containing rabbit anti-Pfs16 antiserum (1:200) and incubated on ice for 30–60 min. Cells were washed three times with FACS buffer and protected from light for all subsequent steps. For secondary staining, Alexa Fluor 488-conjugated goat anti-rabbit IgG (1:500 in FACS buffer) was added, and samples were incubated on ice for 30 min in the dark, followed by three washes with FACS buffer. Nucleic acids were labeled by adding Hoechst (1:1,000 in FACS buffer) for 20 min, and then cells were washed once and resuspended in 100 µL FACS buffer for acquisition. Data were acquired on a flow cytometer equipped for 488-nm (FITC/Alexa Fluor 488) and 405-nm (DAPI/Hoechst) excitation. Gating excluded debris and doublets (FSC/SSC and singlet gates). Counts of Pfs16^+^/DNA^+^ events were obtained for non-induced asexual and gametocytes stages parasites; thresholds were set using the controls below and single-color compensation. Counts of Pfs16^+^/DNA^+^ are provided in Table S5. Statistical analyses were performed using GraphPad Prism (Version 10.4.1).

## Data Availability

Single-cell RNA sequencing data for Plasmodium have been submitted to the Sequence Read Archive (SRA) under accession numbers: SRR36862457 (NF54) and SRR36862456 (LRR5).

## AFTER EPUB

*[This article was published on 15 June 2026 with an error in Prem Prakash's surname. The name was corrected in the current version, posted on 18 June 2026.]*

## SUPPLEMENTAL MATERIAL

Data Set S1 msphere.00186-26-s0001.xlsx Additional data and analyses.

ASM does not own the copyrights to Supplemental Material that may be linked to, or accessed through, an article. The authors have granted ASM a non-exclusive, world-wide license to publish the Supplemental Material files. Please contact the corresponding author directly for reuse.
