# Erratum for Pires et al., “*Plasmodium falciparum* leucine-rich repeat 5 disruption alters the transcription progression during asexual and sexual stage development”

**DOI:** 10.1128/msphere.00592-26

**Published:** 2026-08-26

**Authors:** Camilla V. Pires, Changqi C. Q. Wang, Min Zhang, Chiara Micchelli, Prem Prakash, Jenna Oberstaller, Julian C. Rayner, Thomas D. Otto, John H. Adams

## ERRATUM

Volume 11, no. 7, e00186-26, 2026, https://doi.org/10.1128/msphere.00186-26. Byline: Jenna Oberstaller’s name should appear as shown in this Erratum.

